# A Scientific Hoax

**Published:** 1841-10

**Authors:** 


### A SCIENTIFIC HOAX.

A shower of "*flesh and blood*" was reported to have refreshed the "cedars of Lebanon," in Wilson county, Tennessee, about the middle of last August. Scientific gentlemen from a distance visited the spot where this singular shower descended, and reported, that the matter was veritable fat and muscle, such as might once have made a part of the living body of a hog. The story of the shower rested upon the testimony of some negroes who were at work in a tobacco field, and stated, that they saw the "red cloud" from which it fell "passing rapidly towards the West." The negroes, we have been informed, have since confessed, that this "flesh and blood" storm, like the enchantment of Dulcinea, by Sancho Panza, was a thing got up for their own amusement. The process was to scatter the putrefying carcass of a hog over the tobacco patch, particles of which, in the laboratory of our learned and excellent friend, Prof. Troost, were easily enough recognized as animal. Y.

